# Clinical Assessment of Potential Difference in Motion-Tracking Irradiation for Liver Tumors Using Radixact Synchrony®

**DOI:** 10.7759/cureus.81598

**Published:** 2025-04-02

**Authors:** Wataru Okada, Hiroshi Doi, Keisuke Sano, Rina Muraoka, Shimpei Anami, Takashi Shintani, Masao Tanooka

**Affiliations:** 1 Department of Radiotherapy, Takarazuka City Hospital, Takarazuka, JPN; 2 Department of Radiation Oncology, Kindai University Faculty of Medicine, Osakasayama, JPN

**Keywords:** fiducial marker, hepatic tumors, motion-tracking, radixact synchrony, sbrt (stereotactic body radiotherapy)

## Abstract

This study aimed to advance the precision of motion-managed radiotherapy and provide robust information for clinical practice in terms of necessary margins using Radixact Synchrony® (Accuray, Sunnyvale, USA). A retrospective analysis was conducted on 88 irradiation sessions in nine cases of motion-tracking irradiation for liver tumors. Two gold markers were placed near the tumor, and a planning target volume (PTV) margin of 5 mm was set. The interruption threshold for potential difference (PD) (the statistical prediction value of the three-dimensional (3D) distance error from the predictive model) was set at 3.0 mm during treatment. PDvalues obtained before and during treatment, as well as the number of interruptions, were analyzed. A multiple regression analysis was performed to evaluate the influence of various planning parameters, which were obtained during four-dimensional (4D) treatment planning, on the 95th percentile value of PD (PD_95_). The mean PD_95_ was 2.9 ± 0.6 mm. In the four cases where interruptions occurred, the average ± SD number of interruptions per fraction was 7.5 ± 4.6, 3.6 ± 2.5, 2.5 ± 1.5, and 1.0 ± 1.1, respectively. The coefficient of determination (adjusted R^2^) between PD_95_ and planning parameters was 0.802 (p < 0.01), showing a positive correlation with respiratory motion amplitude (p = 0.017) and a negative correlation with inter-marker distance (p = 0.049). No treatment interruptions were experienced in 83.0% of a total of 88 fractions. To conclude, setting a PDinterruption threshold of 3.0 mm for motion-tracking irradiation using Radixact Synchrony® under metallic marker guidance for liver tumors was appropriate for minimizing treatment interruptions with sufficient dosimetric precision. In addition, inter-marker distance and respiratory motion amplitude were associated withPD_95_.

## Introduction

Stereotactic body radiation therapy (SBRT) has emerged as a cornerstone treatment modality for liver tumors and offers a high local control rate through high-dose radiation delivery to small well-defined target volumes [1]. However, the dynamic motion of liver tumors necessitates larger planning target volume (PTV) margins to account for positional uncertainty, which may inadvertently increase the radiation exposure to adjacent organs at risk (OARs) [2]. Dynamic tumor tracking using Radixact Synchrony® (Accuray, Sunnyvale, USA) represents a pivotal advancement in addressing this limitation. By synchronizing beam delivery with real-time tumor motion, these systems enable substantial PTV margin reductions while maintaining dose coverage of the target and sparing critical OARs [3,4].

During real-time tracking of fiducial positions, which are representative of tumor positions, several control parameters that manage the prediction model accuracy are used to enable stable and accurate irradiation applications. One of these control parameters, the potential difference (PD), is a metric that represents the alignment error between the predicted and actual fiducial positions and influences the responsiveness and dosimetric precision of the system. While a stringent PD threshold can improve tracking accuracy and dose conformity, it may also lead to increased treatment interruptions, thus extending the overall beam-on-time and potentially compromising the treatment workflow. Conversely, a relaxed threshold risk degrades tumor coverage and dose distribution. Although the current literature includes several reports on the clinical use and physical verification of irradiation accuracy with the Radixact Synchrony® system [5,6], the optimal PD threshold for liver tumor SBRT that balances these competing considerations remains unexplored. Furthermore, the relationship between PD values and patient-specific characteristics, including respiratory motion amplitude, respiratory cycle, inter-marker distance, and beam-on time, has yet to be systematically investigated.

This study retrospectively evaluated the impact of PD threshold settings on the performance of the Synchrony® system in liver SBRT with fiducial marker tracking. Our objectives were twofold: (i) to assess the feasibility of reducing PTV margins with Synchrony® while maintaining dose distribution reproducibility, and (ii) to identify key factors influencing PD and their implications for treatment interruptions. This investigation aimed to advance the precision of motion-managed radiotherapy and provide robust margin information for using the Radixact Synchrony® in clinical practice.

## Materials and methods

This retrospective study was approved by the Clinical Research Ethics Committee of Takarazuka City Hospital (approval no. 20241003). Written informed consent for radiotherapy was obtained from all patients prior to radiotherapy. Informed consent for this study was obtained using an opt-out form. The study was conducted in accordance with the principles of the Declaration of Helsinki.

Nine patients who received motion-tracking irradiation for liver tumors and a total of 88 irradiations using Radixact Synchrony® that took place between May 2020 and February 2024 were retrospectively analyzed in this study. The CT resolution used in our center was consistently set at 1.37 × 1.37 × 1.0 mm³. In this study, we used a Gold Anchor (GA) (Naslund Medical AB, Huddinge, Sweden) as the fiducial marker. The GA containing 0.5% iron was 0.28 mm in diameter and 10 mm in length and associated with few artifacts; it is not only useful for kilovoltage (kV) planar imaging but also provides excellent visibility with CT and MRI for prostate and liver imaging clinically. Two fiducial markers were implanted at arbitrary positions within 3 cm from the tumor border in each patient. PTV was created by adding 5 mm margins to gross tumor volume (GTV) in all directions. The PD threshold for treatment interruption was set at 3.0 mm. In addition, the respiratory cycle and respiratory motion amplitude were obtained from the respiratory synchronizer system, AZ-733VI (Anzai Medical, Tokyo, Japan), used to acquire the four dimensional (4D) CT, and the beam-on time and inter-marker distance were calculated from the treatment planning system, Precision® (Accuray, Sunnyvale, USA). These parameters were selected to assess their effects on PD. The 95th percentile of PD (PD_95_) during beam-on time was used as the key parameter to assess motion-tracking precision, and treatment interruptions were analyzed based on the PD threshold. All analyses were performed using IBM SPSS Statistics v28.0 (IBM Corp., Armonk, USA) to evaluate the effects of the selected parameters on PD. Statistical significance was determined a priori, with the significance level set at p = 0.05 (2-tailed).

## Results

Actual and planned parameters for each case are shown in Table 1, and the mean PD_95_ was 2.9 ± 0.5 mm across all nine patients and 88 irradiations. Five patients did not experience any interruptions during the treatment, while four patients did. The average number of interruptions per fraction for the remaining four patients (cases 1, 4, 5, and 7) was 7.5 ± 4.6, 3.6 ± 2.5, 2.5 ± 1.5, and 1.0 ± 1.1, respectively (Table 1). Of the 88 irradiations, no treatment interruptions were observed in 73 (83.0 %) fractions.

**Table 1:** Prescription dose and planned parameters for each patient. Number of interruptions per fraction was calculated as the total number of discontinuations during all treatments divided by the number of fractions. PD_95_: 95th percentile of potential difference

Case	Prescription dose	Mean PD_95 _(mm)	Number of interruptions per fraction	Mean respiratory cycle (sec)	Beam-on time (sec)	Respiratory motion amplitude (mm)	Inter-marker distance (mm)
1	40 Gy/4 fr	3.7	3.6 ± 2.5	3.5	418.2	14.8	33.2
2	48 Gy/20 fr	3.3	0.0	4.4	130.2	11.8	28.9
3	40 Gy/5 fr	3.28	0.0	5.5	475.2	14.9	24.9
4	52.5 Gy/15 fr	3.24	2.5 ± 1.5	4.0	213.4	14	16.4
5	50 Gy/5 fr	3.09	7.5 ± 4.6	2.8	645.8	11.8	23.7
6	40 Gy/5 fr	2.7	0.0	3.5	544.6	11.7	35.6
7	50 Gy/5 fr	2.49	1.0 ± 1.1	4.0	449.7	8.4	28.1
8	50 Gy/10 fr	2.39	0.0	3.5	571.7	10.7	38.4
9	40 Gy/10 fr	2.05	0.0	3.6	380.3	10.3	49.6
	Mean ± SD	2.9 ± 0.5	1.6 ± 2.6	3.8 ± 0.8	416.6 ± 166.3	12.1 ± 2.2	30.9 ± 9.7

**Figure 1:**
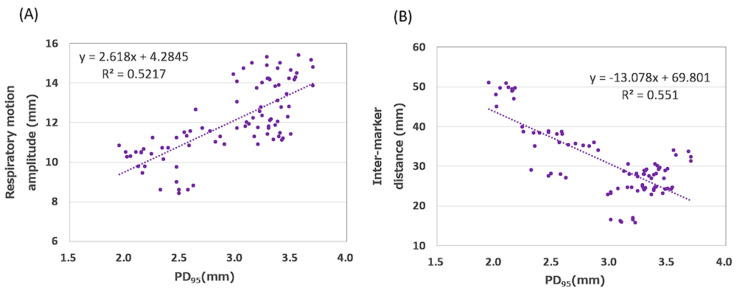
Correlation between PD95 and inter-marker distance, as well as between PD95 and respiratory motion. (A) Respiratory motion amplitude showed a positive correlation with PD_95 _(p = 0.017). (B) The inter-marker spacing exhibited a negative correlation with PD_95_ (p = 0.049). PD_95_: 95th percentile of potential difference

**Figure 2:**
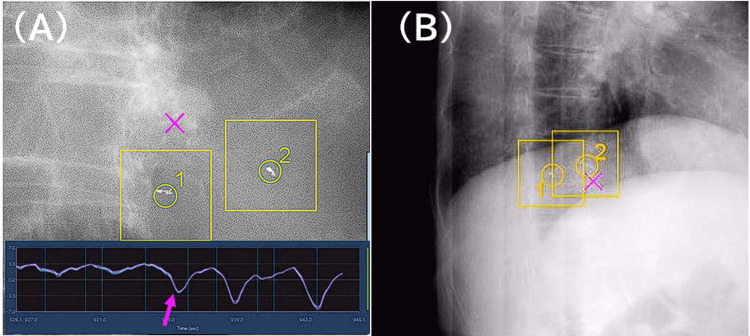
Examples of treatment interruptions. The bottom part of (A) shows the amplitude over time of external respiratory signals obtained from the abdominal wall movement. (A) is an example of an interruption due to a change in breathing pattern at the timing of the arrow (case 5). (B) shows an example where the markers were misrecognized and treatment was interrupted because the markers were too close to each other (case 4).

The multiple regression analysis revealed a coefficient of determination (adjusted R²) of 0.802 (p < 0.01) between PD_95_ and treatment planning parameters (Table 2). Respiratory motion amplitude positively correlated with PD_95_ (p = 0.017), whereas marker spacing negatively correlated with PD_95_ (p = 0.049) (Table 3, Figure 1). These results indicate that larger respiratory motion amplitudes increase PD_95_, whereas a smaller inter-marker distance increases PD_95_. The primary causes of treatment interruption were image recognition errors and irregular breathing patterns. Specifically, cases 5 and 4, as shown in Figures 2A-2B, exhibited frequent interruptions due to irregular breathing patterns and close inter-marker distance, leading to misrecognition. 

**Table 2:** Model summary of the multiple regression analysis of potential factors associated with PD95. The coefficient of determination (adjusted R²) between PD_95_ and the treatment planning parameters was 0.802 (p < 0.01). PD_95_: 95th percentile of potential difference

Observation	F	Prob > F	R^2^	Adjusted R^2^
88	10.1	< 0.01	0.890	0.802

**Table 3:** Results of the multiple regression analysis of potential factors associated with PD95. The respiratory motion amplitude and inter-marker distance showed positive and negative correlations with PD_95_, respectively. PD_95_: 95th percentile of potential difference

Predictor variables	Coefficients	Standard error	t	p	Lower 95%	Upper 95%
Constant	2.515	0.918	2.739	0.041	0.155	4.875
Mean respiratory cycle (sec)	-0.091	0.139	-0.653	0.543	-0.449	0.267
Beam-on time (sec)	-0.001	0.001	-1.94	0.11	-0.002	0
Respiratory motion amplitude (mm)	0.167	0.048	3.498	0.017	0.044	0.289
Inter-marker distance (mm)	-0.025	0.01	-2.572	0.049	-0.051	0

## Discussion

Previous reports have proposed 4 mm isotropic PTV margins that encompass Synchrony® errors and rigid body errors for liver SBRT treatments on CyberKnife® (Accuray, Sunnyvale, USA) [7-9]. The mean PD_95_ across all cases in this study was 2.9 mm, which suggests that the selected threshold of 3.0 mm was feasible and that the conventional 5 mm PTV margin applied in liver SBRT can be reduced. The PTV size is associated with the mean dose to the liver, which can lead to lethal radiation-induced liver damage in SBRT [10]. Radiation-induced gastrointestinal damage is an adverse event that can occur after liver SBRT [11]; thus, a reduction in the PTV margin could improve the safety of liver SBRT in clinical practice. However, a small margin can lead to errors in dose distribution. We have recently suggested a PD threshold of approximately 3.0 mm, from the standpoint of dose distribution reproducibility based on gamma-criteria in phantom studies [6]. In addition, no treatment interruptions were observed in > 80% of fractions. The ability to maintain reproducible dose distributions (gamma pass rate > 90% at 3%/1 mm) with a PD threshold of 3.0 mm, which was adopted in this study, represents a critical balance between dosimetric precision and workflow efficiency.

In this study, the multiple regression analysis identified two key factors, respiratory motion amplitude and inter-marker distance, that significantly influenced PD_95_. As a significant positive correlation was observed between PD_95_ and respiratory motion amplitude, large respiratory excursions affected the predictability of tumor motion. Thus, respiratory motion management is important in tumor motion-tracking SBRT. Conversely, the negative correlation observed between PD_95_ and inter-marker distance suggests that a large distance between fiducial markers provides robust positional data for motion prediction algorithms. In addition, closely spaced fiducial markers can result in misidentification during tracking. Therefore, further studies are needed to assess the optimal distance of fiducial markers in tumor motion-tracking SBRT using Radixact Synchrony®. Moreover, patient-specific observations clarified the primary causes of treatment interruption. In particular, irregular respiratory patterns in two patients disrupted the predictive model, leading to frequent interruptions (cases 1 and 5; Table 1); therefore, respiratory coaching is required to improve the handling of irregular patterns [12].

Treatment interruptions not only prolong beam-on time but may also introduce dose inaccuracies due to intra-fractional motion variability during beam pauses. In contrast, a recent update to the tracking algorithm and X-ray imaging system (from iDMS® system v2.0 to v3.0 (Accuray, Sunnyvale, USA)) has been shown to enhance the system’s responsiveness to changes in the fiducial/chest correlation and to enhance the ability to track targets more accurately [13,14]. Therefore, recently updated systems can help avoid treatment interruptions, even when the same PD threshold settings are used.

Our study has several limitations that should be noted when interpreting our findings, including the equipment and study design. Chan et al. observed larger rotations in a subset of their patients, with 23% of patients having rotations > 2° [9]. Radixact Synchrony® does not allow for target rotation compensation, and such cases must be considered separately. Second, the exclusion of marker-less tracking methods limits the generalizability of the findings to fiducial marker-based approaches. However, marker-less tracking may be less feasible in liver SBRT than in lung SBRT because soft tissue, such as normal liver, exists around the tumor. Marker-less tracking using lipiodol after transarterial chemoembolization should be explored using Radixact Synchrony® [15]. Further comparative studies between Synchrony® and other tracking systems, including marker-less approaches, can help elucidate the advantages and limitations of different motion management strategies. Moreover, while the PD thresholds were set at 3.0 mm based on the gamma analysis in our previous study, additional prospective studies that incorporate clinical outcomes, such as tumor control and toxicities, are needed to validate the clinical appropriateness of this threshold.

## Conclusions

The findings of this study underscore the clinical feasibility and dosimetric benefits of using a PD threshold of 3.0 mm in Synchrony®-based liver SBRT. In addition, the inter-marker distance and respiratory motion amplitude were associated with PD_95_. This study provides a foundation for real-time motion-tracking protocols in clinical practice, thus contributing to the advancement of precision radiotherapy for liver tumors.
